# Review of "clustering for data mining: a data recovery approach" by Boris Mirkin

**DOI:** 10.1186/1475-925X-5-34

**Published:** 2006-05-23

**Authors:** Ritaban Dutta

**Affiliations:** 1 Research Engineer, University of Warwick, Coventry CV4 7AL, UK

I was extremely delighted when when I was invited to review the book by Prof. Boris Mirkin. During my review period I realized once again that the most interesting fundamental feature for a good scientific book should be full of examples, as an example is like a picture which can carry thousand words.

Prof. Mirkin's book on data clustering has 7 chapters all together and 266 pages. After reviewing this book, as a researcher in the field of Intelligent Data mining and Data Clustering I should say that this is a very good and easy reading book with very strong scientific and mathematical material.

In the present world most of the serious decision making problems have similar characteristics, where often we need to consider multiple classification methodologies. Classification-based problem solving approaches need advanced Intelligent Data Mining and Data clustering algorithms. Mathematicians develop Clustering algorithms and engineers use them for real world applications. It is very difficult for an engineer to understand the difficult mathematics behind the algorithms, whereas mathematical developments sometimes do not follow real life application scenarios. So there is a gap which affects the real application-based engineering research, especially in the field of data mining and clustering-based data recovery approach. Prof. Mirkin has done a great job by writing this book in style. Book chapters explain the algorithms for clustering very well, but most interestingly on the basis of pictures and examples. We read this book to learn, perhaps to refresh our knowledge. As an engineer, and not a mathematician, I personally feel that I had a comfortable time during reading this book. This book also covers the historical development of the clustering algorithms for data mining field, its difficulties and limitations, which guided new developments in this field.

Chapter 5 (Data Recovery Models) and Chapter 6 (Different Clustering Approaches) are the most interesting chpaters in this book, as these chapters cover most practical and realistic scientific information. One can read through these chapters keeping the clustering problems in mind and then decide which approach would be best suitable for the particular problem under consideration. They are good coverage of existing data recovery tools and easy to follow and understand. The 'Overall assessment' section at the end of each chapter is a helpful summary to link with the next chapter.

Overall, I should recommend all engineering students and research engineers to read this book, especially those who are doing research in the field of data clustering, classification algorithms development, intelligent signal processing, biomedical and sensory classification problems.

